# Identification of STEAP3-based molecular subtype and risk model in ovarian cancer

**DOI:** 10.1186/s13048-023-01218-x

**Published:** 2023-06-29

**Authors:** Zouyu Zhao, Chongfeng Sun, Jishuai Hou, Panpan Yu, Yan Wei, Rui Bai, Ping Yang

**Affiliations:** 1 First Affiliated Hospital, Shihezi University, Shihezi, China; 2 NHC Key Laboratory of Prevention and Treatment of Central Asia High Incidence Diseases, First Affiliated Hospital, School of Medicine, Shihezi University, Shihezi, China

**Keywords:** Ovarian cancer, STEAP3, Prognostic signature, Immune infiltration

## Abstract

**Background:**

Ovarian cancer (OC) is one of the most common malignancies in women. It has a poor prognosis owing to its recurrence and metastasis. Unfortunately, reliable markers for early diagnosis and prognosis of OC are lacking. Our research aimed to investigate the value of the six-transmembrane epithelial antigen of prostate family member 3 (STEAP3) as a prognostic predictor and therapeutic target in OC using bioinformatics analysis.

**Methods:**

STEAP3 expression and clinical data were acquired from The Cancer Genome Atlas (TCGA), Genotype-Tissue Expression (GTEx), and Gene Expression Omnibus (GEO). Unsupervised clustering was used to identify molecular subtypes. Prognosis, tumor immune microenvironment (TIME), stemness indexes, and functional enrichment analysis were compared between two definite clusters. Through the least absolute shrinkage and selection operator (LASSO) regression analysis, a STEAP3-based risk model was developed, and the predictive effectiveness of this signature was confirmed using GEO datasets. A nomogram was used to predict the survival possibility of patients. Additionally, TIME, tumor immune dysfunction and exclusion (TIDE), stemness indexes, somatic mutations, and drug sensitivity were evaluated in different risk groups with OC. STEAP3 protein expression was detected using immunohistochemistry (IHC).

**Results:**

STEAP3 displayed marked overexpression in OC. STEAP3 is an independent risk factor for OC. Based on the mRNA levels of STEAP3-related genes (SRGs), two distinct clusters were identified. Patients in the cluster 2 (C2) subgroup had a considerably worse prognosis, higher immune cell infiltration, and lower stemness scores. Pathways involved in tumorigenesis and immunity were highly enriched in the C2 subgroup. A prognostic model based on 13 SRGs was further developed. Kaplan-Meier analysis indicated that the overall survival (OS) of high-risk patients was poor. The risk score was significantly associated with TIME, TIDE, stemness indexes, tumor mutation burden (TMB), immunotherapy response, and drug sensitivity. Finally, IHC revealed that STEAP3 protein expression was noticeably elevated in OC, and overexpression of STEAP3 predicted poor OS and relapse-free survival (RFS) of patients.

**Conclusion:**

In summary, this study revealed that STEAP3 reliably predicts patient prognosis and provides novel ideas for OC immunotherapy.

**Supplementary Information:**

The online version contains supplementary material available at 10.1186/s13048-023-01218-x.

## Introduction

Ovarian cancer (OC) is one of the deadliest gynecological malignancies owing to its high recurrence rate and chemoresistance [1]. OC has three different histologic types: epithelial, the most prevalent subtype; germ cell origin; and sex cord-stromal. OC incidence and mortality have decreased significantly in recent decades due to improvements in medical and surgical treatment and the introduction of immune therapeutics [2, 3]. The five-year survival rate is still < 50% in OC [4, 5]. A previous study showed approximately 19,880 new OC cases in the United States in 2022, with 12,810 new OC deaths, accounting for 2.1% and 4% of all new cancer cases and cancer deaths, respectively [1]. Owing to the insidious onset, being prone to invasion and metastasis, and difficulty in early diagnosis, the most majority of OC patients are at an advanced stage when diagnosed [6]. Despite constant advancements in detection and treatment, OC poses a substantial risk to women’s health and is a major societal issue [7]. At present, some widespread blood biomarkers, such as AFP [8], CEA [9], CA199 [10], CA125 [11], HE4 [12], and BRAC1 [13], are employed as diagnostic tools for OC. However, these indicators have not been demonstrated to be the best for precisely determining each patient’s prognosis and curative efficacy [14]. Therefore, identifying specific biomarkers of OC to better understand its progression and develop novel therapeutic targets is essential and urgent.

The six-transmembrane epithelial antigen of prostate family member 3 (STEAP3) was first discovered in prostate tissues as a potential target for prostate cancer immunotherapy [15], also known as tumor suppressor activated pathway-6 (TSAP6). STEAP3 plays a crucial regulatory role in ferroptosis by mediating iron metabolism [16].

A high level of STEAP3 expression supports the proliferation of numerous cancer cells by stimulating iron uptake and preserving iron storage, including glioblastoma [17], hepatocellular carcinoma [18], bladder cancer [19], colorectal cancer [20], etc. Despite the significance of the STEAP gene family in tumorigenesis and development, comprehensive analyses of the importance of STEAP3 in OC remain insufficient.

We performed detailed analyses of STEAP3 in this study in order to clarify its functions and potential mechanisms of action. It has been shown that STEAP3 affected the progression and prognosis of OC, based on several open databases. Through unsupervised clustering and least absolute shrinkage and selection operator (LASSO) regression analysis, molecular typing and prognostic models were constructed based on prognosis-associated STEAP3-related genes (SRGs). We then described the association of risk signatures with tumor immune microenvironment (TIME), tumor immune dysfunction and exclusion (TIDE), stemness indexes, and tumor mutation burden (TMB). Furthermore, the relationship between STEAP3 and the pharmacogenomic features of OC was explored. Finally, we verified the correlation between STEAP3 protein level and prognosis by immunohistochemistry (IHC).

## Materials and methods

### Data download

Gene expression matrix (FPKM), somatic mutation data, and clinical information for OC (n = 378) and normal samples (n = 88) were obtained from the Cancer Genome Atlas (TCGA, https://portal.gdc.cancer.gov/) and Genotype-Tissue Expression (GTEx, http://gtexportal.org/) databases. Moreover, GSE18520, which contained 53 OC samples and 10 normal ovarian tissue samples; GSE19829, which included 28 OC samples; and GSE63885, which consisted of 101OC tissue samples; were obtained from the Gene Expression Omnibus (GEO, https://www.ncbi.nlm.nih.gov/geo/) database. The annotation platform for three datasets was GPL570. The “sva” package was utilized to batch-normalize the expression matrix from the three independent datasets [21].

### Screening SRGs

Patients with OC were divided into two groups according to the median value of the STEAP3 expression. The SRGs between the two groups were determined using the “limma” package. The cutoff criteria were set as |log2 fold change (FC) | > 1.5 and false discovery rate (FDR) < 0.01.

### Cluster analysis

Univariate Cox regression analysis was used to screen out prognosis-related SRGs (p < 0.05). STEAP3-related molecule subtypes were identified by cluster analysis using package “ConsensusClusterPlus” based on the expression of prognosis-related SRGs. Survival analysis was performed to compare the prognosis between the two clusters.

### Immune landscape analysis

Immune cell and immune function activity scores were calculated using single-sample Gene Set Enrichment Analysis (ssGSEA) via “Gene set variation analysis (GSVA)” package. Table S1 described the marker genes and their functions for different immune cells from previous studies [22, 23]. Two immune-related algorithms, including Estimation of stromal and immune cells in malignant tumor tissues using expression data (ESTIMATE) and Cell type identification by estimating relative subsets of RNA transcripts (CIBERSORT), were utilized to analyze the immunological characteristics between cluster 1 (C1) and cluster 2 (C2) by package “IOBR”[24].

### Tumor stemness indexes

The one-class logistic regression (OCLR) algorithm was used to calculate the messenger RNA stemness index (mRNAsi) and epigenetically regulated messenger RNA stemness index (EREG mRNAsi) of each patient with OC based on RNA-seq data of pluripotent stem cell samples from the Progenitor Cell Biology Consortium (PCBC) database [25].

### Enrichment analysis

GSEA was conducted to investigate the differences in signaling pathways activated with the hallmark gene sets and c2kegg gene sets as the reference using GSEA software (version 4.2.3). Annotated gene sets were collected from the Molecular Signatures Database (MSigDB, https://www.gsea-msigdb.org/gsea). The filter criteria were |Normalized enrichment score (NES)|>1, p < 0.05 and FDR < 0.25.

### Construction of the STEAP3-based risk model

LASSO Cox regression was used to identify the potential prognostic SRGs by “glmnet” package [26]. Multivariate Cox analysis was used to determine the optimized risk signature. The formula of the risk model was as follows: risk score = ${\sum }_{\text{k}-1}^{\text{n}} coefi*Expi$, in which coefi indicates the regression coefficients of prognostic SRGs and Expi indicates the expression of genes. Thereafter, on the basis of the median risk score, patients with OC were categorized into high- and low-risk groups. The relationship between the risk score and overall survival (OS) were analyzed using Kaplan-Meier (KM) curves and log-rank test by “survival” package. The prognostic value of the signature was assessed using receiver operating characteristic (ROC) curves and the area under the curve (AUC) through package “timeROC”. Additionally, the risk signature was externally validated using the GEO database.

### Establishment of a nomogram

The independence of risk score was determine using univariate and multivariate Cox analyses combing clinical features, including age, stage, grade, and treatment (pharmaceutical therapy and radiation therapy). The nomogram was established by using package “rms”. Calibration curves, concordance index (C-index) curves and ROC curves were conducted to evaluate the prognostic value of the nomogram for predicting OS [27].

### Immunotherapy response and drug sensitivity

TIDE scores were used to assess each patient’s response to OC immunotherapy [28, 29]. We calculated TIDE related scores on predicting anti-PD1 and anti-CTLA4 response based on the expression matrix of OC (http://tide.dfci.harvard.edu/). In addition, the drug sensitivity of each patient with OC was predicted using the Genomics of Drug Sensitivity in Cancer (GDSC) database (https://www.cancerrxgene.org/). The package “oncopredict” was utilized to calculate the half-maximal inhibitory concentration (IC50) [30].

### Somatic mutations analysis

The package “maftools” was used to calculate the TMB of each patient based on somatic mutation data of OC patients [31]. We then assessed the correlation between the risk score and TMB. KM curve was utilized to compare the differences of OS among different TMB and risk scores groups.

### Human tissue samples

A total of 111 OC and 30 normal fallopian tube epithelial tissue samples were collected from the First Affiliated Hospital of Shihezi University from 2010 to 2022. None of the patients had received chemotherapy, immunotherapy, or radiotherapy prior to specimen collection. All patients had complete clinical and prognostic information. This study was approved by the First Affiliated Hospital of Shihezi University, Shihezi, China (KJX-2021-111-02).

### IHC

First, paraffin-embedded tissue sections were de-waxed in xylene three times for 5 minutes each and rehydrated in graded alcohol. Then these sections were boiled in sodium citrate buffer for antigen retrieval for 8 minutes. The sections were then incubated with 3% hydrogen peroxide for 10 minutes protected from light to block endogenous peroxidase and non-specific binding sites. Tissue sections were incubated with anti-STEAP3 polyclonal antibody (1:100 dilution, Rabbit, Thermo Fisher Scientific) at 4 ℃ overnight. The tissue sections were incubated with biotin-labeled anti-rabbit secondary antibody for 30 minutes at 37 ℃. Finally, 3,3’-diaminobenzidine (DAB) chromogenic solution was used to visualize for 3 min and hematoxylin was utilized to counterstain for 30 s. The staining intensity score (no staining = 0, light brown = 1, brown = 2, and dark brown = 3) and staining area (0–5% = 0, 6–25% = 1, 26–50% = 2, 51–75% = 3, and 76–100% = 4) were multiplied to obtain an IHC score. The criteria used were as follows: 0–6 = weak positive, and > 6 = strong positive.

### Statistical analysis

Statistical analyses were performed using R software (4.2.1). Wilcoxon test was used to compare differences of means between the two groups. The chi-squared test was used to analyze the correlation between STEAP3 levels and clinical parameters and the relationship between immunotherapy response and risk scores. KM curves and log-rank tests were used for survival analysis. Calibration curves, C-index curves and ROC curves were used to evaluate the predictive accuracy of risk models and nomogram models. Univariate and multivariate Cox analyses were performed to assess whether STEAP3 is an independent prognostic factor for OS and relapse-free survival (RFS). P < 0.05 was significant differences. *p < 0.05; **p < 0.01; ***p < 0.001.

## Results

### Expression and prognostic value of STEAP3 in OC

**Fig. 1:**
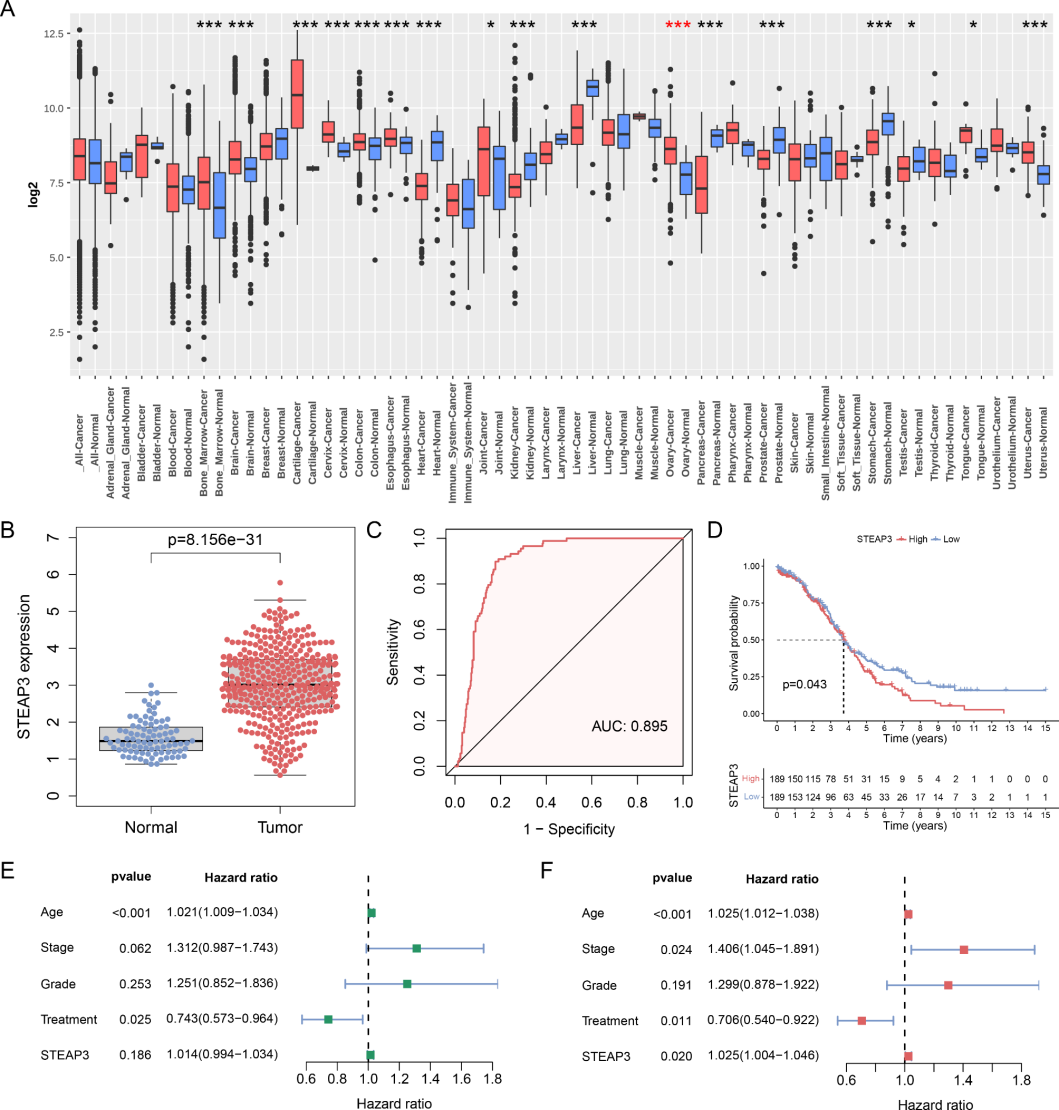
The expression and prognosis of STEAP3 in OC. (**A**) GENT2 database explored differential expression of STEAP3 between multiple cancers and matched normal samples. (**B**) The overexpression of STEAP3 in OC (n = 378) compared with normal group (n = 88) for TCGA and GTEx databases. (**C**) Diagnostic ROC curve of STEAP3. (**D**) KM analysis of OS between two groups. Correlation between OS and clinical parameters including STEAP3 in OC by (**E**) univariate and (**F**) multivariate cox analysis. *p < 0.05; **p < 0.01; ***p < 0.001

In the Gene Expression database of Normal and Tumor tissues (GENT2) database, STEAP3 mRNA expression was remarkably elevated in most malignancies compared to matched normal tissues containing OC (Fig. 1A). STEAP3 was also significantly upregulated in OC from TCGA and GTEx databases (Fig. 1B). The diagnostic efficiency of STEAP3 was evaluated using ROC curves. As shown in Fig. 1C, STEAP3 showed preferable diagnostic performance in distinguishing OC samples from normal samples (AUC: 0.895). KM analysis revealed a worse outcome for patients with high STEAP3 expression (Fig. 1D). STEAP3, age stage and treatment were independent prognostic markers for OC through multivariate Cox analyses (Fig. 1E, F).

### Correlation of molecular subtypes based on prognosis-related SRGs with TIME and stemness

**Fig. 2:**
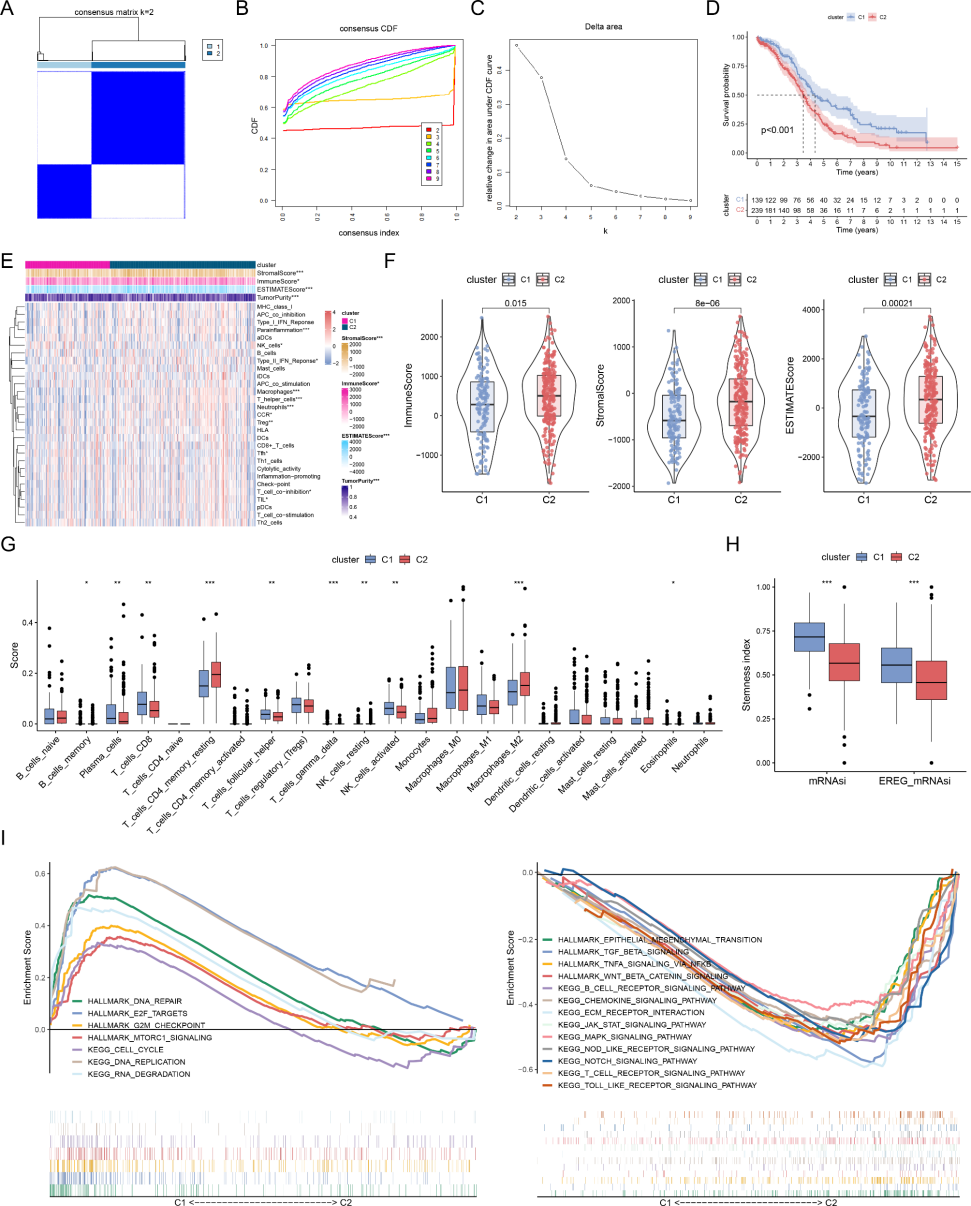
Correlation of molecular subtypes with TIME and stemness. (**A**) Consensus clustering matrix when k = 2. (**B**) Consensus clustering CDF with k valued 2 to 9. (**C**) CDF delta area curve for k = 2. (**D**) Survival curve of OS between two clusters. (**E**) Immune cells infiltration of distinct subtypes by ssGSEA algorithm. (**F**) Differences of immune, stromal and ESTIMATE scores of two subtypes. (**G**) Immune cells components in different subtypes through CIBERSORT. (**H**) Differences of stemness index between two clusters. (**I**) GSEA analyses for SRGs of two clusters. *p < 0.05; **p < 0.01; ***p < 0.001

We identified 1327 SRGs between the different STEAP3 expression groups (Table S2). In total, 222 SRGs were identified to be the prognostic factors influencing the outcomes of patients with OC from the TCGA database (Table S3). These prognosis-related SRGs were then subjected to clustering analysis. Unsupervised clustering was utilized to classify patients with OC into two distinct subtypes (C1 and C2) using the “ConsensusClusterPlus” package (Fig. 2A-C). KM survival analysis revealed a worse prognosis of C2 than C1 (Fig. 2D). In addition, the TIME landscape was determined using various algorithms for the two clusters. Significant differences in the immune cells and immune functions were observed (Fig. 2E). The results of the ESTIMATE algorithm showed that C2 had higher immune, stromal, and ESTIMATE scores (Fig. 2F). As shown in Fig. 2G, the CIBERSORT algorithm revealed that C1 was significantly enriched in several anti-tumor immune cells, such as CD8 T cells, activated natural killer (NK) cells, and T follicular helper (Tfh) cells. These immune cells could inhibit the growth of tumor cells and increase sensitivity to immune checkpoint blockade (ICB) therapy [32]. Immunosuppressive cells, such as resting CD4 memory T cells, resting NK cells, and M2 macrophages, were strongly enriched in the C2 subgroup. M2 macrophages directly promoted the metastasis and chemoresistance of OC cells through secreting a variety of cytokines, chemokines, enzymes, and exosomes [33].

Next, the stemness indexes between C1 and C2 subtypes were compared. The results revealed that the mRNAsi and EREG mRNAsi scores in C1 were remarkably higher (Fig. 2H). This suggests that patients with C1 had a higher proportion of stem cells. Nevertheless, some studies demonstrated that higher stemness was positively correlated with poor prognosis; therefore, further research is needed. Using GSEA enrichment analysis, we explored the biological differences between the two molecular subtypes. The results revealed that immune-related biological processes (B cell receptor signaling pathway, chemokine signaling pathway, and T cell receptor signaling pathway.) and tumorigenic pathways (epithelial-mesenchymal transition, TNFA signaling via NF-kB and notch signaling pathway.) were enriched in the C2 subtype (Fig. 2I). Lin et al. revealed that periostin (POSTN) enhanced M2 macrophages through integrin-mediated NF-κB singling to promote OC metastasis [34]. Special AT-rich sequence-binding protein 1 (Satb1) derived cancer-associated dendritic cells (DCs) differentiation by activating NOTCH1 signaling to regulate major histocompatibility complex class II (MHC II) expression [35].

### Construction and validation of STEAP3-based risk signature for OC prognosis

**Fig. 3:**
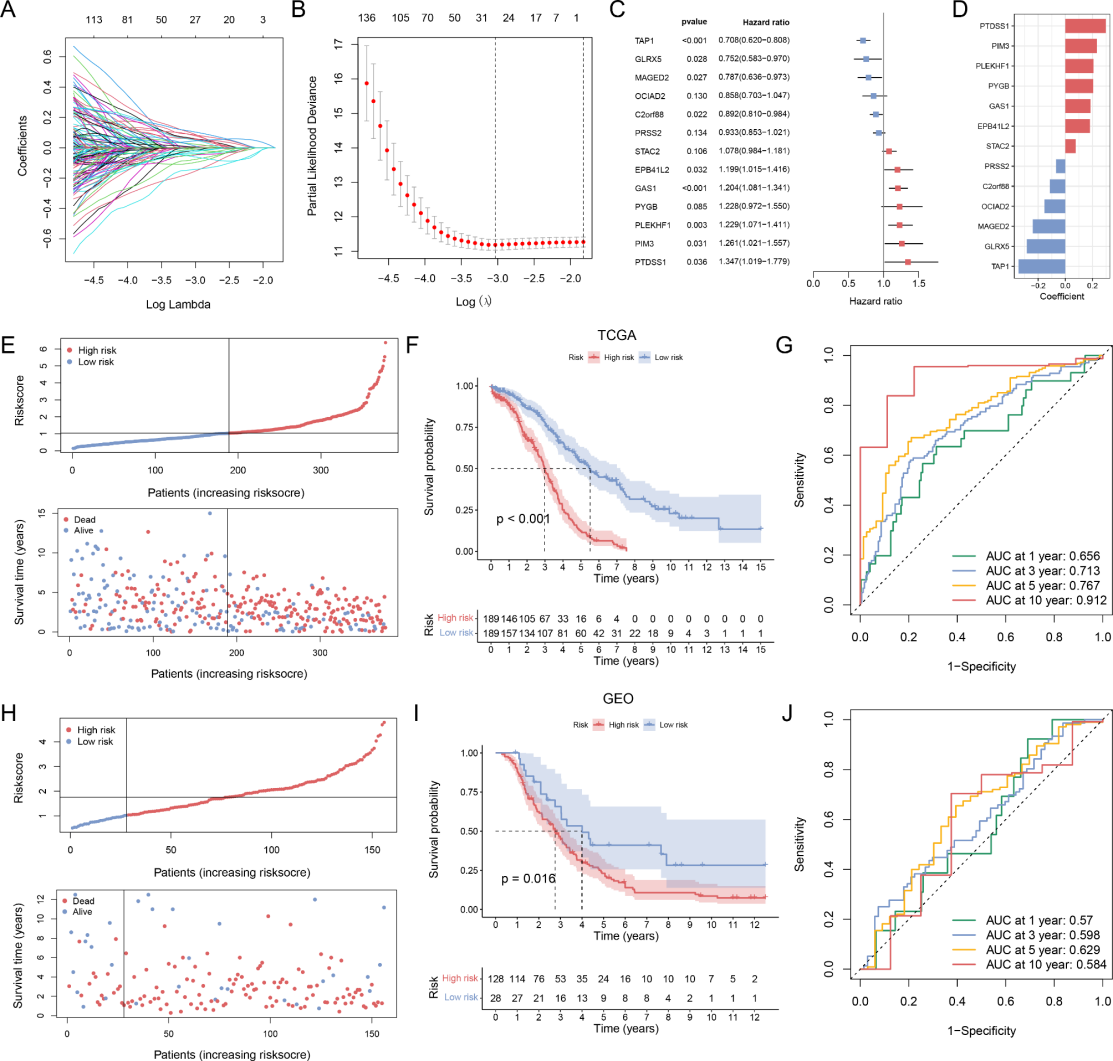
Risk signature based on prognosis-related SRGs. (**A**, **B**) LASSO cox regression analysis of TCGA dataset. (**C**) Multivariate cox regression analysis of thirteen SRGs. (**D**) Coefficients of the 13-gene signature. Distribution of the risk score, survival time and status for (**E**) TCGA cohort and (**H**) GEO cohort. KM curves for (**F**) TCGA cohort and (**I**) GEO cohort. ROC analysis of the risk signature for (**G**) TCGA cohort and (**J**) GEO cohort

First, the LASSO algorithm was implemented to select important candidate genes of the risk signature (Fig. 3A, B). The model achieved optimum performance when λ = 0.01731. The forest plot of multivariate analysis was displayed in Fig. 3C. Finally, the STEAP3-based risk signature was constructed by selecting 13 prognosis-related SRGs. The formula for risk signature was as follows: risk score = 0.181399814 * EPB41L2 + 0.205009036 * PYGB + (-0.23965905) * MAGED2 + 0.075209836 * STAC2 + (-0.15368987) * OCIAD2 + 0.297560054 * PTDSS1 + 0.206376774 * PLEKHF1 + (-0.345589371) * TAP1 + 0.185644738 * GAS1 + (-0.284750657) * GLRX5 + (-0.113831054) * C2orf88 + 0.231985638 * PIM3 + (-0.069014815) * PRSS2. The coefficients of the SRGs are shown in Fig. 3D. A comparison of the expression of these genes in OC and normal tissues can be seen in **Figure **S1.

In the TCGA cohort, patients with OC were classified into two groups (high-risk and low-risk groups) with a medium risk score value. There was a significant increase in mortality risk among patients in the high-risk group compared to patients in the low-risk group (Fig. 3E). According to the KM survival analysis, patients at high risk had shorter survival times than those at low risk (Fig. 3F). Time-dependent ROC curve analysis was used to evaluate the predictive power of the risk score in the TCGA cohort (Fig. 3G). The overall predictive ability and accuracy were quite satisfactory (AUC > 0.65); in particular, the 10-year survival rate showed the greatest predictability and accuracy (AUC: 0.912). Therefore, the risk score is more accurate for predicting long-term outcomes in patients with OC. To verify the predictive performance of the signature, we tested this model using the GEO database (Fig. 3H-J). This result indicates that we constructed an excellent risk model for OC prognosis.

### Establishment of a nomogram combined with clinical parameters

**Fig. 4:**
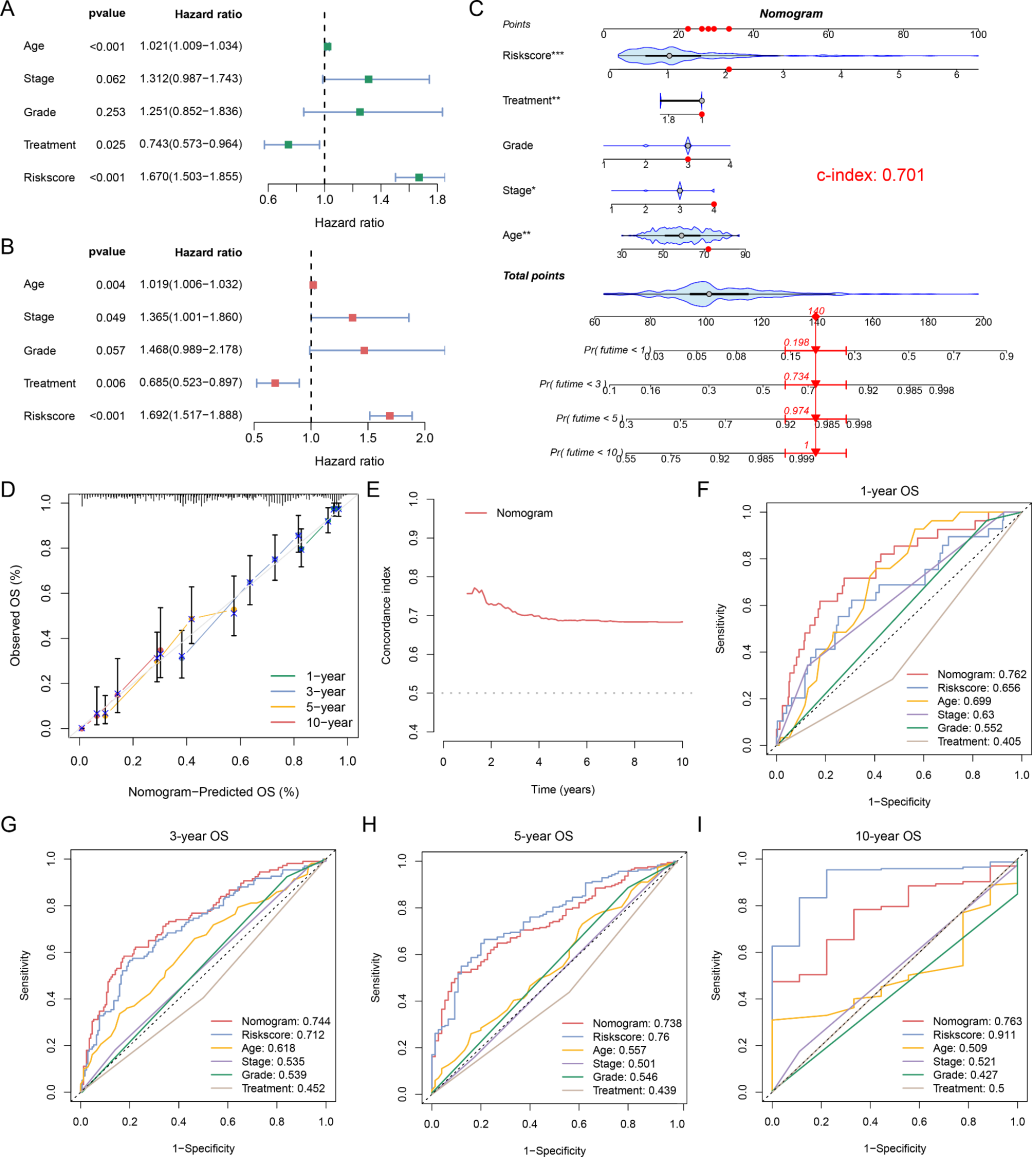
Nomogram model combined with risk score and clinical parameters. (**A**) Univariate and (**B**) multivariate cox regression analysis. (**C**) Nomogram integrated the age, stage, grade, treatment, and risk score. (**D**) Calibration curves of nomogram. (**E**) C-index curve of the nomogram. ROC curves for predicting (**F**) 1-, (**G**) 3-, (**H**) 5-, and (**I**) 10-year OS. *p < 0.05; **p < 0.01; ***p < 0.001

Univariate analysis revealed a remarkable association between age, stage, grade, treatment, and STEAP3-based risk score and OS in OC (Fig. 4A). Among them, age, stage, treatment, and risk score were independent prognostic factors for OC (Fig. 4B). To broaden the clinical application and usability of the STEAP3-based risk model, a nomogram was constructed combing with common clinical parameters that could easily predict survival outcomes of patients with OC (Fig. 4C). The calibration curves suggested that the nomogram performed well (Fig. 4D). In addition, the C-index curve showed that the nomogram had desirable efficacy for predicting patient outcomes (Fig. 4E). By comparing the nomogram, risk score, and clinical parameters, we found that the nomograms were more effective in predicting short-term prognosis (less than 5 years) of patients with OC (Fig. 4F, G). However, the predictive efficiency of the risk score for long‐term outcomes (5- and 10-year) was slightly better compared with the nomogram (Fig. 4H, I).

### Analysis of immune landscape and chemotherapy sensitivity between different risk groups

**Fig. 5:**
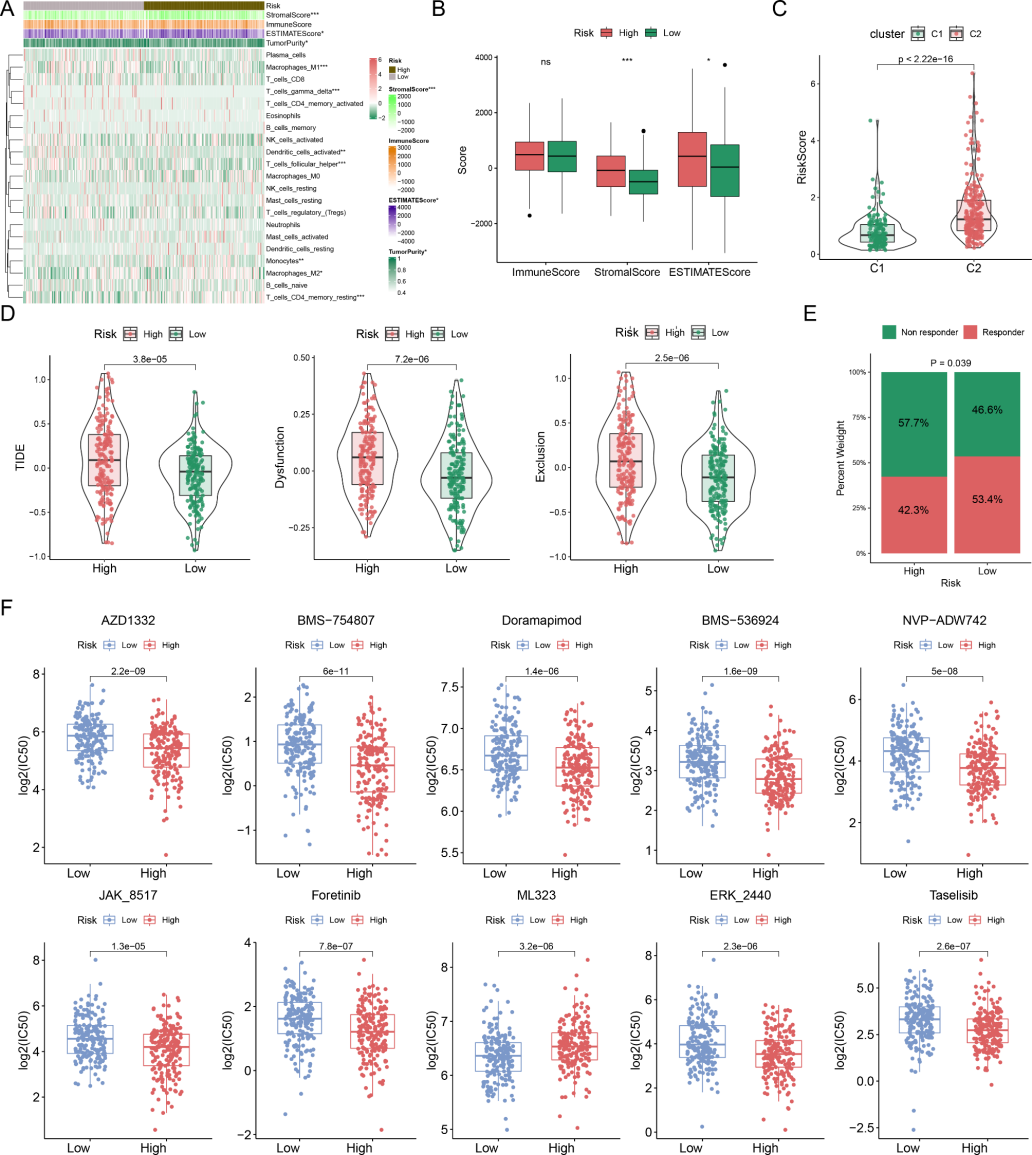
Analysis of immune landscape and chemotherapy sensitivity in different risk groups. (**A**) Comparisons of immune cell infiltration between two risk groups. (**B**) Differences of immune, stromal and ESTIMATE scores of high- and low-risk groups. (**C**) Relationship between the risk score and molecular subtypes. (**D**) Differences of TIDE, T cell dysfunction and T cell exclusion scores in distinct risk groups. (**E**) Distributions of non-responder and responder to immunotherapy between different risk groups. (**F**) Differences of chemotherapy sensitivity between two risk groups. *p < 0.05; **p < 0.01; ***p < 0.001

We further explored the correlation between the risk model and TIME, and the abundances of 22 immune cells were calculated using the CIBERSORT algorithm. As shown in Fig. 5A, several immunosuppressive cells, including resting CD4 memory T cells and M2 macrophages, were activated in the high-risk group. The abundances of Tfh cells, gamma delta T cells, M1 macrophages, and activated dendritic cells in the low-risk group were significantly higher than those in the high-risk group. The results of the ESTIMATE analysis revealed that the stromal and ESTIMATE scores were observably higher in the high-risk group than in the low-risk group, while the immune score between the two risk groups was not significantly different (Fig. 5B). Next, we investigated the relationships between the risk model and molecular subtypes in OC. A higher risk score was observed for the C2 subtype (Fig. 5C). Subsequently, we used TIDE to evaluate the potential clinical efficacy of the immunotherapy. In the TCGA cohort, TIDE, T cell dysfunction, and T cell exclusion scores in the high-risk group were markedly higher than those in the low-risk group (Fig. 5D). These results indicated that high-risk patients were less likely to benefit from immunotherapy. Furthermore, 42.3% of patients in the high-risk group were estimated to benefit from immunotherapy, which was lower than the low-risk group (53.4%) (Fig. 5E). Surgery and standard chemotherapy combined with carboplatin and paclitaxel are the basic treatment strategies for primary OC [36]. Thus, the IC50 values of the top ten chemotherapy drugs that associated with the risk score were calculated (Fig. 5F). High-risk patients had greater sensitivity to AZD1332, BMS-754,807, Doramapimod, BMS-536,924, NVP-ADW742, JAK_8517, Foretinib, ERK_2440, and taselisib, suggesting that the risk score could be regarded as a potential predictor of chemical sensitivity. Overall, this result demonstrates why patients in the high-risk group have a poor prognosis and a poor response to immunotherapy.

### Correlation of risk score with stemness and TMB

**Fig. 6:**
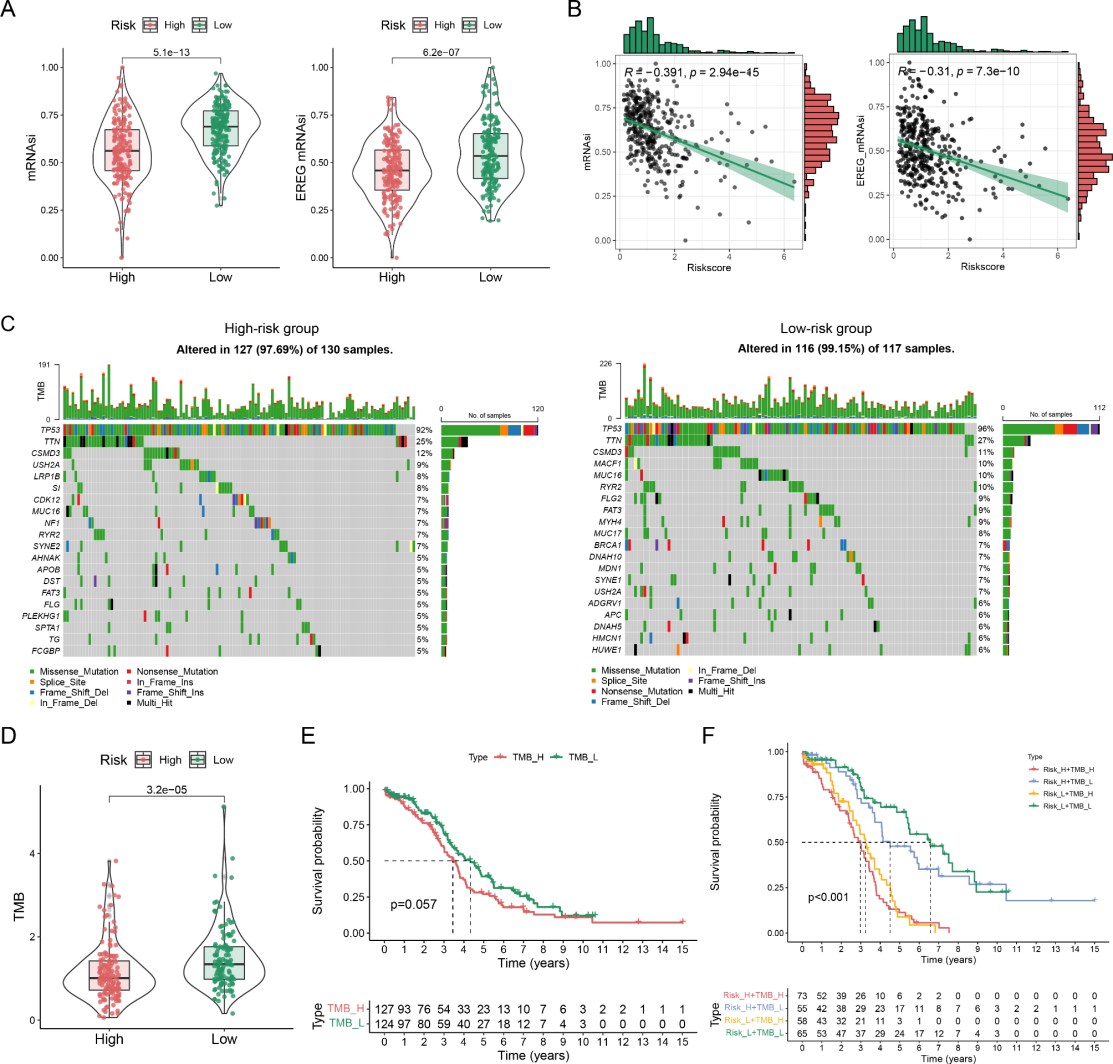
Correlation of risk score with stemness and TMB. (**A**) Differences of stemness index in distinct risk groups. (**B**) Correlation between risk score and stemness index. (**C**) Waterfall maps of the somatic mutations in high- and low-risk groups. (**D**) Differences of TMB between the two groups. (**E**) Survival curve between high and low TMB groups. (**F**) Survival curve based on risk score and TMB.

We compared the differences of stemness indexes between the two risk subgroups and discovered that the mRNAsi and EREG mRNAsi scores were markedly higher in the low-risk subgroup (Fig. 6A) and negatively associated with the risk score (Fig. 6B). In addition, waterfall plots depicted the frequency of mutations in the top 20 genes in the distinct risk groups (Fig. 6C). For instance, TP53 was the most frequently mutated gene in both groups. Somatic mutation analysis indicated that the higher TMB was discovered in the low-risk subgroup (Fig. 6D). Nevertheless, survival analysis showed no obvious differences between the two groups (Fig. 6E). Patients with OC were divided into four groups based on risk score and TMB for further survival analysis. Patients with high TMB or high risk had shorter OS than those with low TMB and low risk (Fig. 6F).

### Correlation between STEAP3 protein level and clinical features

**Fig. 7:**
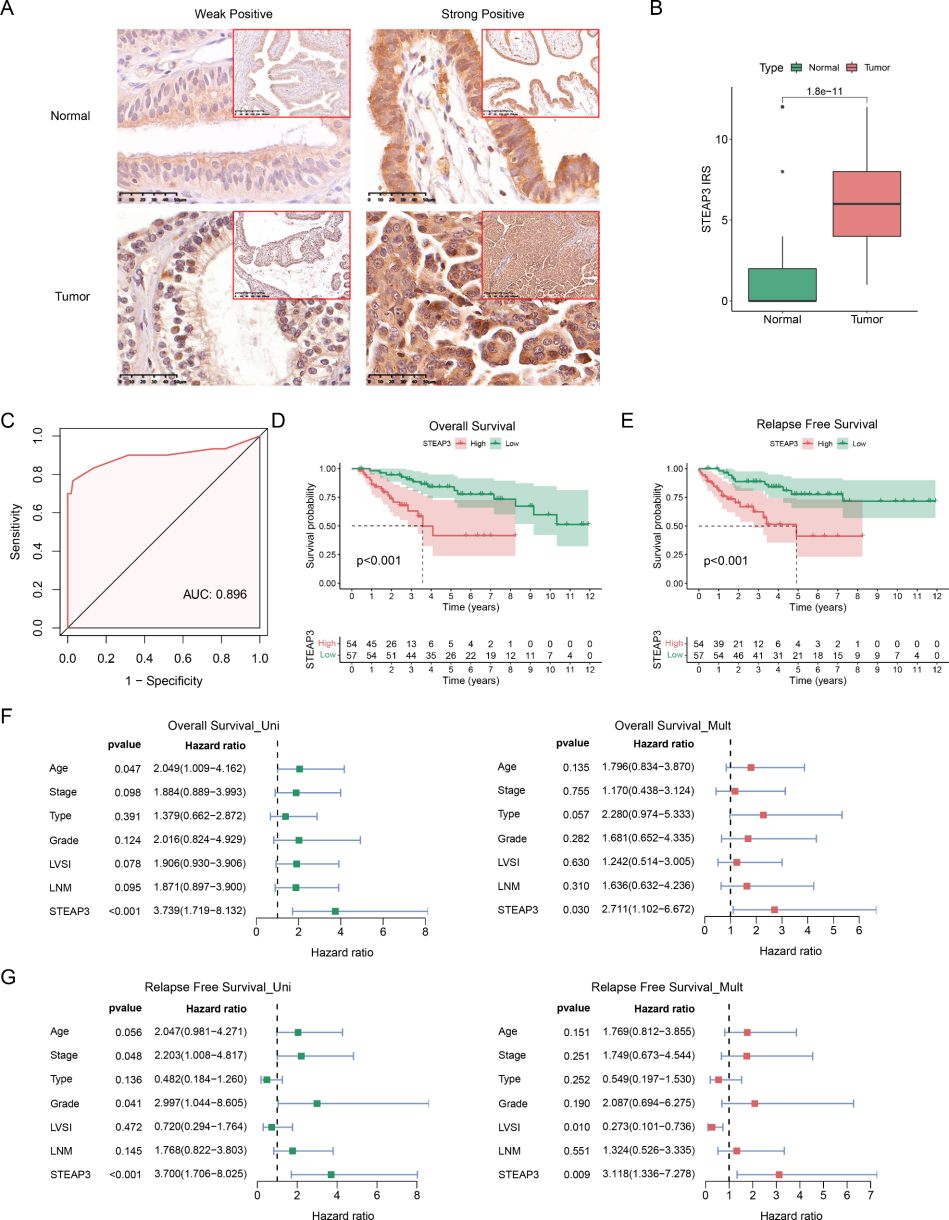
High expression of STEAP3 is correlated with poor outcomes in OC patients. (**A**) The expression of STEAP3 in normal (n = 30) and OC (n = 111) samples were detected by IHC. (**B**) The STEAP3 IRS in normal tissues and OC. (**C**) Diagnostic ROC curve of STEAP3. KM curves for (**D**) OS and (**E**) RFS between different STEAP3 groups. Univariate and multivariate cox analysis of STEAP3 expression and clinical parameters for (**F**) OS and (**G**) RFS in OC.

The STEAP3 protein expression in 111 OC and 30 normal samples was detected using IHC (Fig. 7A). The findings demonstrated that STEAP3 was upregulated in OC tissues (p = 1.8e-11; Fig. 7B). The ROC curve showed that STEAP3 had a significant diagnostic performance for OC (AUC: 0.896, Fig. 7C). We then explored the correlation between STEAP3 expression and the clinicopathological features of OC (Table 1). STEAP3 was significantly associated with age, stage, grade, lymphovascular space invasion (LVSI), and lymph node metastasis (LNM) in patients with OC. Furthermore, KM curve was utilized to investigate the predictive value of STEAP3 for prognosis in OC. The results demonstrated that with an increase in STEAP3 expression, the probability of OS and RFS for patients with OC decreased (Fig. 7D, E). STEAP3 was found to be an independent risk factor by comparing age, stage, type, grade, LVSI, and LNM for OS (Fig. 7F) and RFS (Fig. 7H) of OC according to multivariate analysis.

**Table 1:** Relationship between clinicopathologic characteristics and STEAP3 protein expression in OC.

Characteristics	N	STEAP3, n (%)		χ^2^	P value
		Low	High		
Age (year)					
≤ 52	60	36 (60.0)	24 (40.0)	3.910	0.048
> 52	51	21 (41.2)	30 (58.8)		
Stage					
I-II	52	37 (71.2)	15 (28.8)	15.356	< 0.001
III-IV	59	20 (33.9)	39 (66.1)		
Grade					
Low	30	20 (66.6)	10 (33.3)	3.860	0.049
High	81	37 (45.7)	44 (54.3)		
Type					
Serous carcinoma	78	38 (48.7)	40 (51.3)	0.728	0.393
Others	33	19 (57.6)	14 (42.4)		
LVSI					
Negative	84	49 (58.3)	35 (41.7)	6.739	0.009
Positive	27	8 (29.6)	19 (70.4)		
LNM					
Negative	80	49 (61.3)	31 (38.8)	11.235	0.001
Positive	31	8 (25.8)	23 (74.2)		

LVSI, lymphovascular space invasion; LNM, lymph node metastasis.

## Discussion

It has been proven that STEAP3 overexpression is involved in tumor progression and predicts poor outcomes in several types of cancer [18, 37, 38]. However, the molecular mechanisms and oncogenic roles of STEAP3 remain unclear. This study comprehensively analyzed the prognostic values, immune infiltration patterns, and therapeutic responses of STEAP3 across OC. Our findings demonstrated that STEAP3 expression was noticeably upregulated in OC. STEAP3 overexpression predicted poor outcomes in patients with OC. Furthermore, STEAP3 was an independent prognostic biomarker for OC using multivariate analysis.

Precise molecular subtyping could be a novel strategy to guide more effective patient-specific treatments. The C2 subtype in this study was characterized by aberrant enrichment of prognosis-related SRGs with poorer prognosis and higher tumor-promoting cell infiltration, such as resting memory CD4 T cells, resting NK cells, and M2 macrophages, and higher immune and stromal scores. The results of ssGSEA showed that C2 subtype had the higher abundance of NK cells and lower abundance of Tfh cells. NK cells detected the loss of Human Leukocyte Antigen (HLA) by killing targets through antigen-independent pathways, exhibiting anti-tumor immune evasion and recurrence functions [39]. The silencing of Stab1 promoted Tfh cells differentiation and resulting the generation of tertiary lymphoid structures (TILs), which was correlated with positive prognosis [40].

GSEA indicated that immune-related and tumorigenesis-related pathways, including epithelial-mesenchymal transition (EMT) and Wnt beta-catenin signaling, were significantly enriched in the C2 group. Recent studies revealed that Wnt activity is an important regulator of EMT [41] and exerts a remarkable role in regulating tumor stemness and chemoresistance in OC [42–44]. These findings partially explain the poor prognosis in the C2 subgroup.

We constructed a STEAP3-based risk model comprising 13 SRGs to determine the prognosis of OC. Several genes in this risk model have been investigated in previous studies. Menyhárt et al. found that EPB41L2 was a biomarker of poor prognosis and topotecan resistance in OC [45]. In triple-negative breast cancer, NOTCH4 transcriptionally upregulated SLUG and GAS1 to maintain mesenchymal-like characteristics of breast cancer stem cells [46]. GAS1, a stemness-related gene, was strongly expressed and predicted poor outcomes in patients with OC [47]. PIM3 was overexpressed, promoting the proliferation and migration in OC [48]. In addition, the clinical outcomes of OC patients were predicted more powerfully and accurately using a nomogram. Consistently, the C2 subgroup had a higher risk score, which was positively related to poorer prognosis for patients with OC.

TIME is closely correlated with tumorigenesis, recurrence, and metastasis [49, 50]. Tumors can shape the TIME into an immunosuppressive state to evade immune surveillance [51]. Therefore, understanding the TIME is crucial for evaluating the effects of immunotherapy. By immune infiltration analysis, we observed that some anti-tumor immune cells, like Tfh cells, gamma delta T cells, M1 macrophages, and activated dendritic cells, were strongly enriched in the low-risk subgroup, whereas tumor-promoting cells, such as M2 macrophages and resting CD4 memory T cells, were more prevalent in the high-risk subgroup. Previous researches shown that lower levels of M1 macrophages or higher levels of M2 macrophages are prognostic risk factors for patients with OC [52, 53]. Macrophages promoted the expression of CXC chemokine ligand 9 (CXCL9) and M1 phenotype via activating the NF-κB signaling pathway, thereby increasing cytotoxic T cell infiltration, and inhibiting the progression of epithelial ovarian cancer [54]. Previous studies showed that tumor association macrophage (TAM) was an important driver tumor metastasis and played a key role in regulating EMT [55]. Gamma delta T cells can eliminate OC cells [56] proliferation, reduce tumor burden, and increase the sensitivity of SKOV3 sphere cells to chemotherapeutic agents by promoting IL17 production [57]. The differences in TIME in distinct risk subgroups might reflect the different benefits of ICB treatment by the TIDE algorithm. The TIDE signature integrates T-cell dysfunction in CTL-high tumors and T-cell exclusion in CTL-low tumors, which model two mechanisms of immune escape [29]. A high TIDE score is correlated with poor immunotherapy response and short survival time of patients. This is consistent with our findings. Patients in high-risk subgroup had a high TIDE score and a poor prognosis, indicating that high-risk individuals benefited less from immunotherapy.

Cancer stem cells are key to tumor initiation, progression, recurrence, metastasis, and drug resistance [58]. The stemness indexes (mRNAsi and EREG mRNAsi) are measures of stem cell characteristics. Our study demonstrated that mRNAsi score was negatively related to risk score, which contradicts our results and requires further study. TMB, a biomarker of immunotherapy, may predict patient survival after ICB treatment [59, 60]. TMB was significantly higher in the low-risk group than the high-risk group. The survival time for the two TMB groups did not differ significantly. TMB, combined with a risk model, could result in greater predictive power for patient survival.

Our study has several limitations. First, the expression matrix and clinical information were downloaded from public databases. We needed a prospective clinical trial cohort to verify the accuracy of the risk model. Second, further functional assays and molecular mechanisms are required to detect the 13 prognosis-related genes. Third, more clinical information is needed regarding the accuracy of the risk score in predicting the response to immunotherapy. Finally, the functional role of STEAP3 in tumor immunity requires in-depth experimental verification.

## Conclusion

In summary, our results demonstrate that STEAP3 could be used as a reliable biomarker for prognosis and immunotherapy.

## Electronic supplementary material

Below is the link to the electronic supplementary material.

**Supplementary Material 1**: Table S1 The marker genes of different immune cells and functions.

**Supplementary Material 2**: Table S2 Identification of SRGs.

**Supplementary Material 3**: Table S3 Univariate cox regression analysis of SRGs.

**Supplementary Material 4**

## Data Availability

The datasets used in this study is available from the corresponding author upon reasonable request.
