# Supplementary material for: Identification of STEAP3-based molecular subtype and risk model in ovarian cancer
Source: J Ovarian Res. 2023 Jun 29;16:126. doi: 10.1186/s13048-023-01218-x (PMC10308644; doi:10.1186/s13048-023-01218-x)
Supplement: Supplementary file 4 — Supplementary Material 4 [file 13048_2023_1218_MOESM4_ESM.pdf]

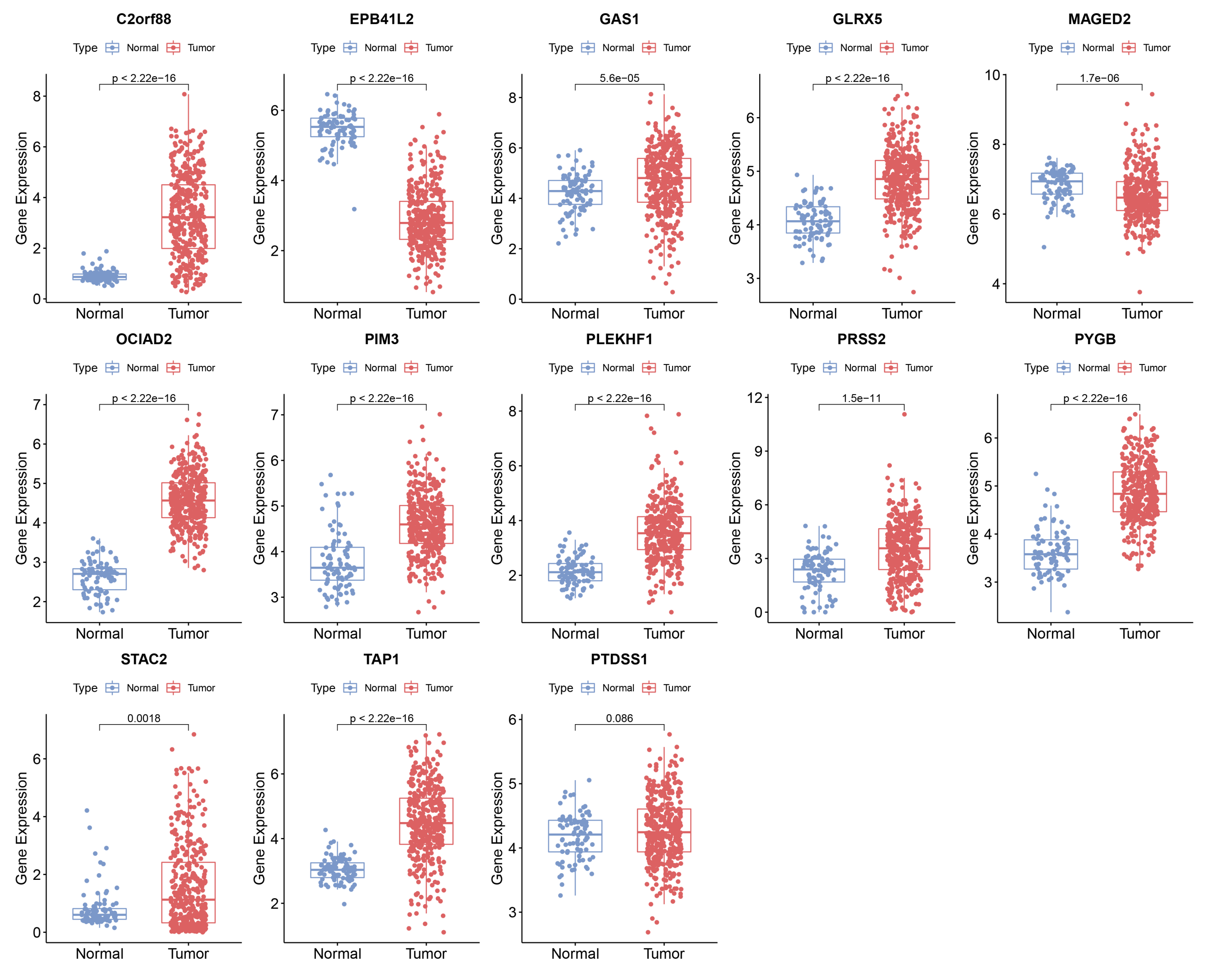
C2orf88
Type Normal Tumor
p < 2.22e−16
Gene Expression
Normal Tumor
EPB41L2
Type Normal Tumor
p < 2.22e−16
Gene Expression
Normal Tumor
GAS1
Type Normal Tumor
5.6e−05
Gene Expression
Normal Tumor
GLRX5
Type Normal Tumor
p < 2.22e−16
Gene Expression
Normal Tumor
MAGED2
Type Normal Tumor
1.7e−06
Gene Expression
Normal Tumor
OCIAD2
Type Normal Tumor
p < 2.22e−16
Gene Expression
Normal Tumor
PIM3
Type Normal Tumor
p < 2.22e−16
Gene Expression
Normal Tumor
PLEKHF1
Type Normal Tumor
p < 2.22e−16
Gene Expression
Normal Tumor
PRSS2
Type Normal Tumor
1.5e−11
Gene Expression
Normal Tumor
PYGB
Type Normal Tumor
p < 2.22e−16
Gene Expression
Normal Tumor
STAC2
Type Normal Tumor
0.0018
Gene Expression
Normal Tumor
TAP1
Type Normal Tumor
p < 2.22e−16
Gene Expression
Normal Tumor
PTDSS1
Type Normal Tumor
0.086
Gene Expression
Normal Tumor
